# Somatic mutation spectrum of a Chinese cohort of pediatrics with vascular malformations

**DOI:** 10.1186/s13023-023-02860-w

**Published:** 2023-09-01

**Authors:** Bin Zhang, Rui He, Zigang Xu, Yujuan Sun, Li Wei, Li Li, Yuanxiang Liu, Wu Guo, Li Song, Huijun Wang, Zhimiao Lin, Lin Ma

**Affiliations:** 1 Department of Dermatology, Beijing Children’s Hospital, Capital Medical University (National Center for Children’s Health, China), No. 56 Nanlishi Road, Xicheng District, Beijing, 100045 China; 2 Department of Dermatology, Zhengzhou University, Affiliated Children’s Hospital, Henan Children’s Hospital, Zhengzhou Children’s Hospital, Zhengzhou, 450000 Henan China; 3 Dermatology Hospital, Southern Medical University, No.2 Lujing Road, Guangzhou, 510091 China

**Keywords:** Somatic mutation, Vascular malformations, Children, PIK3CA, GNAQ

## Abstract

**Background:**

Somatic mutations of cancer driver genes are found to be responsible for vascular malformations with clinical manifestations ranging from cutaneous birthmarks to life-threatening systemic anomalies. Till now, only a limited number of cases and mutations were reported in Chinese population. The purpose of this study was to describe the somatic mutation spectrum of a cohort of Chinese pediatrics with vascular malformations.

**Methods:**

Pediatrics diagnosed with various vascular malformations were collected between May 2019 and October 2020 from Beijing Children’s Hospital. Genomic DNA of skin lesion of each patient was extracted and sequenced by whole-exome sequencing to identify pathogenic somatic mutations. Mutations with variant allele frequency less than 5% were validated by ultra-deep sequencing.

**Results:**

A total of 67 pediatrics (33 males, 34 females, age range: 0.1–14.8 years) were analyzed. Exome sequencing identified somatic mutations of corresponding genes in 53 patients, yielding a molecular diagnosis rate of 79.1%. Among 29 PIK3CA mutations, 17 were well-known hotspot p.E542K, p.E545K and p.H1047R/L. Non-hotspot mutations were prevalent in patients with PIK3CA-related overgrowth spectrum, accounting for 50.0% (11/22) of detected mutations. The hotspot GNAQ p.R183Q and TEK p.L914F mutations were responsible for the majority of port-wine stain/Sturge–Weber syndrome and venous malformation, respectively. In addition, we identified a novel AKT1 p.Q79K mutation in Proteus syndrome and MAP3K3 p.E387D mutation in verrucous venous malformation.

**Conclusions:**

The somatic mutation spectrum of vascular malformations in Chinese population is similar to that reported in other populations, but non-hotspot PIK3CA mutations may also be prevalent. Molecular diagnosis may help the clinical diagnosis, treatment and management of these pediatric patients with vascular malformations.

**Supplementary Information:**

The online version contains supplementary material available at 10.1186/s13023-023-02860-w.

## Introduction

Vascular malformations represent a spectrum of rare disorders resulting from aberrant development of blood and/or lymphatic vessels [1]. The clinical manifestations are quite diverse, ranging from single cutaneous birthmarks to life-threatening systemic anomalies. A uniform classification for vascular malformations has been proposed by the International Society for the Study of Vascular Anomalies (ISSVA), which greatly facilitates the diagnosis, treatment and management of the disorders [2]. Vascular malformations are usually congenital and many mainly present as skin lesion in the early life. The malformations, especially those involving multiple organs and systems, may cause severe clinical problems as the affected child grows up, such as chronic pain, coagulation disorder and organ dysfunction, with increasing disability rate and mortality [3, 4].

In recent years, a set of cancer driver genes have been found to be associated with vascular malformations [5, 6]. Somatic mutations in TEK encoding TEK receptor tyrosine kinase are identified in sporadic venous malformation (VM) and blue rubber bleb nevus syndrome (BRBNS) [7, 8]. Activating mutations in PIK3CA which encodes the phosphatidylinositol-4,5-bisphosphate 3-kinase catalytic subunit alpha cause VM without a TEK mutation [9] and isolated lymphatic malformations (LM) [10]. Moreover, PIK3CA mutations are responsible for a group of disorders characterized by segmental overgrowth of various tissues with or without vascular malformations, which are also termed as PIK3CA-related overgrowth spectrum (PROS) [11]. This spectrum includes DCMO (diffuse capillary malformation with overgrowth) [12], Klippel–Trenaunay syndrome (KTS) [10], CLAPO (capillary malformation of the lower lip, lymphatic malformation of the face and neck, asymmetry and partial/generalized overgrowth) [13], CLOVES (congenital lipomatous overgrowth, vascular malformations, epidermal nevi, and skeletal/spinal abnormalities) [14], megalencephaly-capillary malformation (MACP) [15], and other entities. GNAQ, encoding the G protein subunit alpha q, is somatically mutated in isolated capillary malformation termed as port-wine stain (PWS) and the more severe neurocutaneous Sturge–Weber syndrome (SWS) that is characterized by facial port-wine stains, and ocular and cerebral vascular malformations [16, 17]. The other disease-causing genes includes AKT1 for Proteus syndrome [18], MAP3K3 for verrucous venous malformation (VVM) [19], GNA11 for DCMO [20], RASA1 and EPHB4 with germline or somatic mutations for capillary malformation-arteriovenous malformation (CM-AVM) [21, 22], and so on. Somatic mutations in these genes aberrantly activate signaling pathways, such as PI3K/ATK/mTOR and Ras/Raf/MEK/ERK pathways, that are responsible for cell proliferation, differentiation, migration and apoptosis, leading to vascular and/or lymphatic hyperproliferation and tissue overgrowth [5, 23].

Till now, dozens of mutations have been identified in patients with vascular malformation and the mutation spectrum has been well delineated. However, most studies on large case series were performed in countries different from China [24–26]. Only a limited number of vascular malformations with a definitive pathogenic mutation were previously reported in Chinese population [27–29]. There is a lack of study describing the somatic mutation spectrum in a large cohort of Chinese patients. Here, we reported the genetic findings of a cohort of Chinese children with various vascular malformations who mainly referred to hospital due to dermatological abnormalities, aiming to provide evidence for the early molecular diagnosis of this group of patients.

## Methods

### Participants

Between May 2019 and October 2020, consecutive patients who were referred to the Department of Dermatology, Beijing Children’s Hospital affiliated to Capital Medical University, Beijing were recruited. The inclusion criteria were as follows: (1) patients aged < 18 years; (2) patients were diagnosed with specific vascular malformation classification according to ISSVA criteria [2]; (3) the patients’ parents and/or the patients gave written informed consent to participate in the research. This study was approved by the Ethic Committee of Beijing Children’s Hospital.

### Whole-exome sequencing

We collected peripheral blood and biopsy of skin lesion from each patient included in our study, and separated skin lesion into epidermis and dermis after an incubation with 0.25% dispase II (Roche) 4 °C overnight as described previously [30]. Genomic DNA were extracted from dermis and blood samples by using corresponding DNA extraction kits. Whole-exome sequencing (WES) was performed by using DNA from skin lesions. Briefly, the exome was captured by GenCap Human Exome Kit (MyGenostics Co.Ltd., Beijing, China) and the sequencing library was constructed according to the manufacturer’s instruction. Massively parallel sequencing was conducted on Illumina NexSeq 500 platform. After quality control of raw data, the sequencing reads were mapped to the human reference genome (hg19) by using Burrows-Wheeler Aligner (BWA 0.7.10). Single nucleotide variants (SNVs) and insertion/deletions (indels) were detected by GATK (4.0.8.1) HaplotypeCaller and annotated by ANNOVAR (http://www.openbioinformatics.org/annovar/).

Since most of vascular anomalies were caused by postzygotic mutations in a set of genes, we focus on somatic nonsynonymous mutations in known disease-causing genes corresponding to specific vascular anomaly classification. A somatic mutation should be covered by at least 5 identical variant alleles with > 1% variant allele frequency (VAF) that was determined by the number of variant reads divided by the total number of mapping reads. The number of a mutation identified in cancers and general population was retrieved from Catalogue Of Somatic Mutations In Cancer (COSMIC v95, https://cancer.sanger.ac.uk/cosmic/) and Genome Aggregation Database (gnomAD v2.2.1, http://gnomad.broadinstitute.org), respectively. In silico prediction on the mutations was performed by using SIFT (http://provean.jcvi.org/), PolyPhen2 (http://genetics.bwh.harvard.edu/pph2/) and MutationTastor (http://www.mutationtaster.org).

### Validation of somatic mutations

All identified disease-causing somatic mutations were firstly verified by manually scanning the mapping alignment by Integrative Genomics Viewer (IGV, http://software.broadinstitute.org/software/igv/igvtools) to exclude misalignment. Then, Sanger sequencing in peripheral blood DNA was performed to exclude germline mutation. Furthermore, mutations with less than 5% VAF were validated by ultra-deep sequencing in skin lesion which yielded thousands of mapping reads. In brief, each mutation was amplified by polymerase chain reaction (PCR) using specific primers, ligated to sequencing adaptors, and then sequenced on a next-generation sequencing (NGS) platform. Bioinformatic analysis detecting the mutation was the same as the aforementioned method in WES.

### Statistics

Categorical variables were presented as percentages and compared by Fisher’s exact test. Continuous variables were presented as median with interquartile range (IQR) and range and compared by Mann–Whitney rank sum test. All statistics were performed by using GraphPad Prism 8. *P* < 0.05 indicated statistical significance.

## Results

### Overall molecular diagnosis

A total of 67 pediatrics diagnosed with vascular anomalies were included, of which 33 (49.3%) were males and 34 (50.7%) were females. The median age was 2.3 years (IQR: 0.7–6.0; range 0.1–14.8), and 21 (31.3%) patients were under the age of 1 year (Table 1). The clinical diagnosis included 4 CLOVES, 15 KTS, 1 unclassified PROS (uPROS), 3 DCMO, 6 capillary lymphatic venous malformation (CLVM), 2 capillary-lymphatic malformation (CLM), 1 lymphatic malformation (LM), 7 capillary malformation with overgrowth (CMO), 12 PWS, 2 SWS, 6 venous malformation (VM), 7 verrucous venous malformation (VVM) and 1 Proteus syndrome.

**Table 1:** Characteristics of pediatric patients with vascular malformation

Variable	N, (%)	Molecular diagnosis rate, N (%)
Total	67	53 (79.1)
Sex		
Male	33 (47.8)	24 (72.7)
Female	34 (52.2)	29 (85.3)
Median age, years	2.3	–
IQR	0.7–6.0	–
Range	0.1–14.8	–
Age distribution		
<1 year	21 (31.3)	16 (76.2)
1–3 years	17 (25.4)	15 (88.2)
3–10 years	22 (34.3)	18 (81.8)
>10 years	7 (9.0)	4 (57.1)
Diagnosis		
CLOVES	4 (6.0)	4 (100)
KTS	15 (22.4)	14 (93.3)
uPROS	1 (1.5)	1 (100)
DCMO	3 (4.5)	0 (0)
CLVM	6 (9.0)	5 (83.3)
CLM	2 (3.0)	1 (50.0)
LM	1 (1.5)	1 (100)
CMO	7 (10.4)	6 (85.7)
PWS	12 (17.9)	10 (83.3)
SWS	2 (3.0)	2 (100)
VM	6 (9.0)	5 (83.3)
VVM	7 (10.4)	3 (42.9)
Proteus syndrome	1 (1.5)	1 (100)

*KTS*: Klippel–Trenaunay syndrome; *SWS*: Sturge–Weber syndrome; *DCMO*: diffuse capillary malformation with overgrowth; *VM*: venous malformation; *CLOVES*: congenital lipomatous overgrowth, vascular malformations, epidermal nevi, scoliosis/skeletal and spinal syndrome; *PWS*: port-wine stains; *VVM*: verrucous venous malformation; *CMO*: capillary malformation with overgrowth; *LM*: lymphatic malformation; *CLVM*: capillary lymphatic venous malformation; *uPROS*: unclassified PIK3CA-related overgrowth spectrum; *CLM*: capillary-lymphatic malformation

**Fig. 1:**
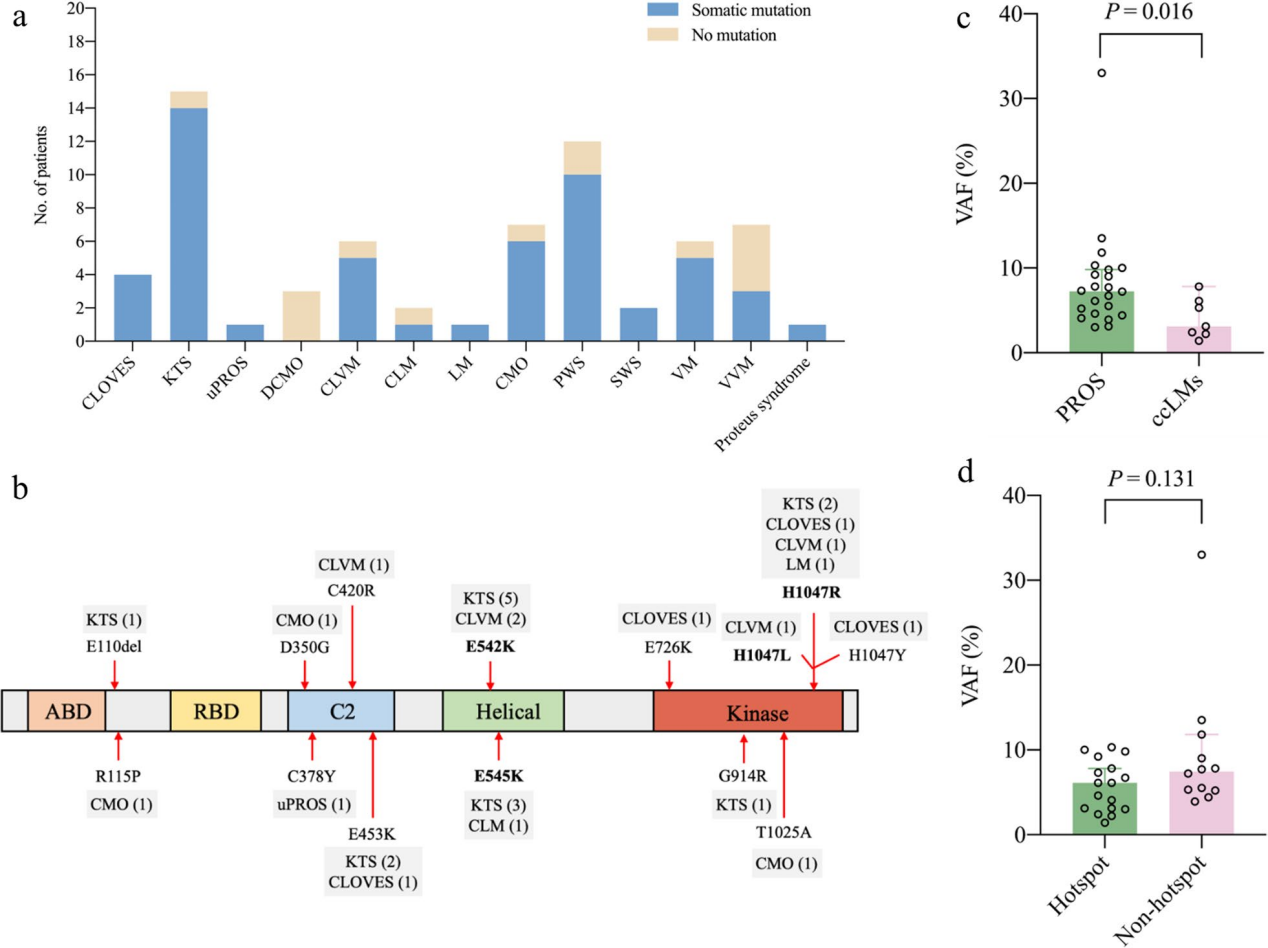
Molecular diagnosis in pediatrics with vascular malformations by whole-exome sequencing. **a** Molecular diagnosis yield in vascular malformations. **b** Distribution diagram of PI3KCA somatic mutations. **c** VAF of PIK3CA mutations in patients with PROS and ccLMs. **d** VAF of PIK3CA hotspot and non-hotspot mutations. CLM: capillary-lymphatic malformation; CLOVES: congenital lipomatous overgrowth, vascular malformations, epidermal nevi, scoliosis/skeletal and spinal syndrome; CLVM: capillary lymphatic venous malformation; CMO: capillary malformation with overgrowth; DCMO: diffuse capillary malformation with overgrowth; KTS: Klippel–Trenaunay syndrome; LM: lymphatic malformation; PWS: port-wine stains; SWS: Sturge–Weber syndrome; uPROS: unclassified PIK3CA-related overgrowth spectrum; VAF: variant allele frequency; VM: venous malformation; VVM: verrucous venous malformation

Whole-exome sequencing identified pathogenic somatic mutations in corresponding disease-causing genes in 53 out of 67 patients, yielding an overall molecular diagnosis rate of 79.1% (Additional file 1: Table S1). The median VAF was 6.3% (IQR: 4.0–7.8; range: 1.4–33.0). The diagnosis yield varied among different malformations (Fig. 1a). All CLOVES (4/4), uPROS (1/1) and almost all KTS cases (14/15) and CLVM cases (5/6) had PIK3CA somatic mutations. All SWS (2/2) and the majority of PWS (10/12) harbored GNAQ somatic mutations. TEK somatic mutations were found in 5 of 6 VM cases, and MAP3K3 mutations were identified 3 of 7 VVM patients. Among 7 CMO patients, 3 had PIK3CA mutations and 3 carried GNA11/Q mutations. None of DCMO (0/3) were molecularly diagnosed. The molecular diagnosis rate did not differ between male and female and among different age groups. All somatic mutations have been verified by manually scanning the reads alignment by IGV tools, and excluded for germline change by direct sequencing in peripheral blood. Furthermore, 18 mutations with < 5% VAF of WES methods were validated by ultra-deep sequencing except 7 mutations that were not determined due to insufficient DNA samples (Additional file 1: Table S1).

### PROS and non-syndromic LMs

PIK3CA-related malformations can be divided as overgrowth syndromes caused by PIK3CA mutations which are typically called PROS, and non-syndromic common and combined LMs (ccLMs) [31]. In our study, there were 30 PROS patients who were diagnosed with CLOVES, KTS, DCMO, CMO or uPROS, and 9 ccLMs cases diagnosed with LM, CLM or CLVM. We identified 14 different PIK3CA activating mutations in 29 pediatrics, including p.E110del, p.R115P, p.D350G, p.C378Y, p.C420R, p.E453K, pE542K, p.E545K, p.E726K, p.G914R, p.T1025A, p.H1047Y, p.H1047R and p.H1047L (Fig. 1b). The overall molecular diagnosis rate of PIK3CA-related malformations was 74.4%. 22 of 30 PROS patients and 7 of 9 ccLMs patients were found to harbor a PIK3CA somatic mutation. Thus, both groups had a similar molecular diagnosis rate (*P* = 0.789). The median VAF of PIK3CA mutations was 6.1% (IQR: 4.1–9.0; range: 1.4–33.0). VAF was higher in PIK3CA-mutated PROS patients than ccLMs (Fig. 1c), and did not differ between hotspot and non-hotspot mutations (Fig. 1d).

**Fig. 2:**
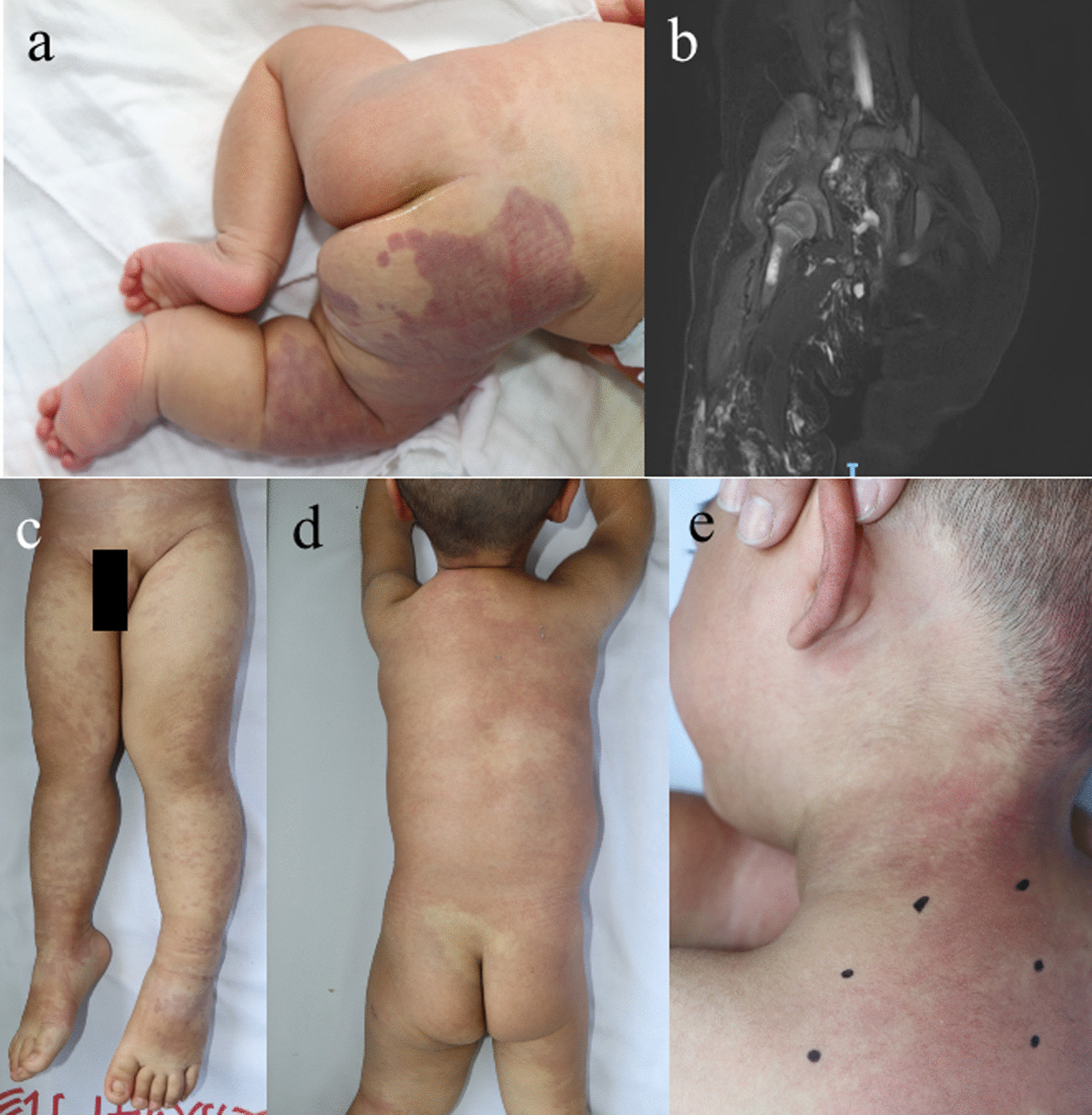
Pediatric PROS patients harboring PIK3CA somatic non-hotspot mutations. **a** Patient P53, a 1-month-old girl with KTS and PIK3CA c.328_330delGAA;p.E110del mutation. She had right lower limb and foot overgrowth. **b** T2 phase of MRI showed multiple streaky vascular shadows with hypersignal pressure on subcutaneous soft tissue of buttock and proximal femur. **c** Patient P83, a 20-month-old boy with CLOVES and PIK3CA c.2176G > A;p.E726K mutation. He had left lower limb overgrowth, **d** subcutaneous mass on the left back, **e** and epidermal nevi on the neck

In present study, PIK3CA somatic driver mutations distributed across the whole gene, but more than half (17/29, 58.6%) were at hotspot positions, including p.E542K, p.E545K and p.H1047R/L, that had strong oncogenic activity [32] (Table 2). The hotspot p.E542K were identified in 5 KTS and 2 CLVM, and p.E545K were found in 3 KTS and 1 CLM. The p.H1047R/L mutation was harbored by 2 KTS, 2 CLVM, 1 uPROS and 1 LM. On the other hand, the non-hotspot mutations collectively contributed to the development of vascular malformations in a considerable proportion of patients (12/29, 41.4%). Out of the 22 mutations detected in PROS, 50.0% (11/22) were located in non-hotspot residues, which was similar to 48.0% (12/25) of a previous report [31]. On the contrary, only 1 of 7 PIK3CA-mutated ccLMs had non-hotspot mutations, but the proportion of non-hotspot mutations did not differ between PROS and ccLMs (Fisher’s exact test, *P* = 0.187). Despite moderate or unknown oncogenic activity, these non-hotspot activating mutations also caused typical capillary malformations on both trunks and extremities and overgrowth of extremities in pediatrics (Fig. 2). Medical imaging showed thickened soft tissue with increased blood vessels in overgrown extremities (Fig. 2).

**Table 2:** Summary of somatic mutations identified in pediatric patients with vascular malformation

Gene	Mutation	Oncogenic activity ^a^	COSMIC ^b^	gnomAD ^c^	Present study ^d^	Hotspot
PIK3CA (NM_006218)	c.328_330delGAA;p.E110del	Unknown	41	0	1	No
	c.344G > C;p.R115P	Unknown	6	0	1	No
	c.1049A > G;p.D350G	Unknown	10	0	1	No
	c.1133G > A;p.C378Y	Unknown	5	0	1	No
	c.1258 T > C;p.C420R	Strong	216	0	1	No
	c.1357G > A;p.E453K	Intermediate	88	0	3	No
	c.1624G > A;p.E542K	Strong	1887	0	7	Yes
	c.1633G > A;p.E545K	Strong	3064	1	4	Yes
	c.2176G > A;p.E726K	Unknown	160	0	1	No
	c.2740G > A;p.G914R	Unknown	6	0	1	No
	c.3073A > G;p.T1025A	Unknown	54	0	1	No
	c.3139C > T;p.H1047Y	Intermediate	117	0	1	No
	c.3140A > G;p.H1047R	Strong	3786	1	5	Yes
	c.3140A > T;p.H1047L	Strong	565	1	1	Yes
AKT1 (NM_005163)	c.235C > A;p.Q79K	–	22	0	1	No
GNA11 (NM_002067)	c.547C > T;p.R183C	–	24	0	2	Yes
GNAQ (NM_002072)	c.547C > G;p.R183G	–	4	0	1	No
	c.548G > A;p.R183Q	–	50	0	12	Yes
TEK (NM_000459)	c.2740C > T;p.L914F	–	0	0	5	Yes
MAP3K3 (NM_002401)	c.1161A > C;p.E387D	–	0	0	1	No
	c.1323C > G;p.p.I441M	–	0	0	2	Yes

^a^According to Dogruluk T’s study (ref. 32) for PIK3CA mutations; ^b^Number of cancer patients with somatic mutation in COSMIC v95 (released 24-NOV-2021); ^c^Number of alleles in gnomAD v2.2.1; ^d^Number of patients with somatic mutation in present study; COSMIC: Catalogue of Somatic Mutations in Cancer (https://cancer.sanger.ac.uk/cosmic/); gnomAD: Genome Aggregation Database (https://gnomad.broadinstitute.org)

In addition to PIK3CA mutations, GNA11/Q mutations are also found to cause CMO [44]. Among 7 CMO in our cohort, 3 harbored PIK3CA non-hotspot mutations (p.R115P, p.D350G and p.T1025A), 2 had GNA11 p.R183C hotspot and 1 carried a rare GNAQ p.R183G mutation. These patients showed capillary malformations limited to one anatomical region and overgrowth of upper limbs (Additional file 2: Figure S1).

In our cohort, one patient (P119) presented diffuse capillary malformation and right upper limb overgrowth (Additional file 3: Figure S2), and also had venous malformation on right upper limb as indicated by MRI. Since the patient had a PIK3CA somatic mutation by exome-sequencing, she was therefore given a diagnosis of unclassified PROS (uPROS). Besides, 3 patients had diffuse capillary malformations distributed on multiple anatomical regions and overgrowth of lower limbs, without venous or lymphatic anomalies by MRI (Additional file 4: Figure S3). Thus, they were diagnosed with DCMO. However, we failed to identify somatic mutations in PIK3CA or GNA11 through exome-sequencing in these patients.

### PWS and SWS

**Fig. 3:**
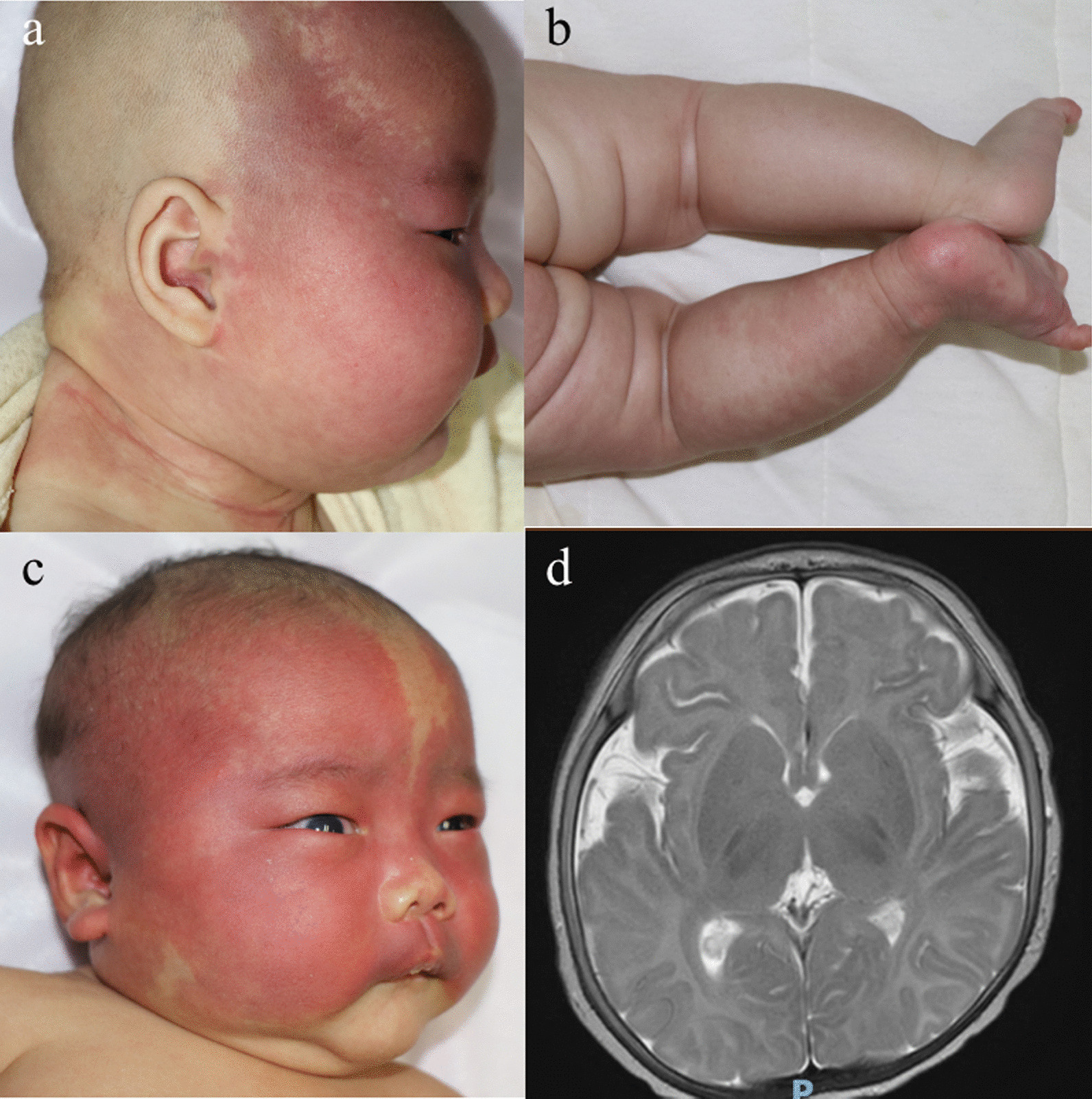
Clinical features of patients with PWS and SWS. **a** Patient P44, a 3-month-old girl with PWS and GNAQ c.548G > A;p.R183Q mutation. She had capillary malformations on the righ face and neck. She had normal intraocular pressure and no abnormalities in cerebral MRI. **b** Patient P41, a 3-month-old girl with PWS and GNAQ c.548G > A;p.R183Q mutation. She showed capillary malformation on the left lower limb and had symmetric low limbs. **c** Patient P36, a 2-month-old boy with SWS and GNAQ c.548G > A;p.R183Q. He had capillary malformation on bilateral lateral parts, right auricle and scalp with clear border, as well as pressure of fading and right eyeball protrusion. **d** MRI showed that outside the brain interval of bilateral temporal lobe were widened significantly with increased vascular shadows, and that some of them were circuity and dilated

This group of patients were mainly characterized by capillary malformation (PWS), with ocular and cerebral vascular anomalies (SWS). We identified GNAQ p.R183Q hotspot in 10 of 12 PWS and in both 2 SWS patients. PWS patients had capillary malformations on faces, limbs and trunks without extracutatneous manifestations such as glaucoma, overgrowth or neurological abnormalities (Fig. 3a, b). In addition to vascular birthmarks, GNAQ mutations may cause ocular and ceberal abnormalities which were defined as SWS (Fig. 3c, d).

### VM

**Fig. 4:**
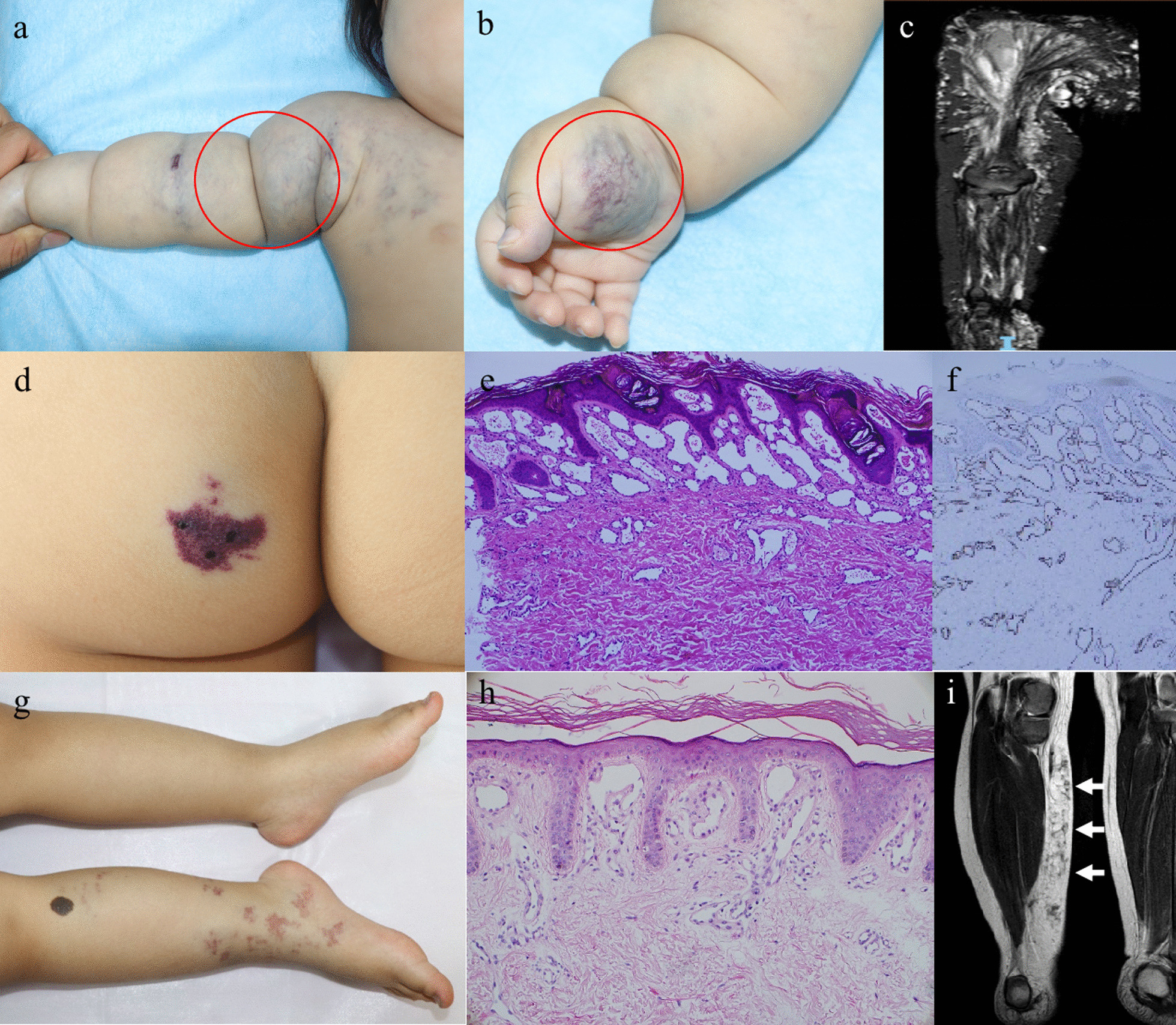
Venous malformation with TEK p.L914F hotspot mutation, and VVM patients with MAP3K3 somatic mutations. **a** Patient P33, a 6-month-old girl, had blue-purple patches on his right upper limb, wall of the chest, neck and back, with tortuous and expanded blue and red blood vessels seen in some areas. **a, b** A blue soft tumor (red circle) can be found on the right underarm and palm thenar, respectively. **c** Coronal reconstruction of MRI showed diffuse high signal shadow in the right upper limb and right shoulder, involving subcutaneous fat and surrounding ulna and radius. **d** Patient P75, a girl at the age of 2 years and 5 months, had MAP3K3 p.I441M mutation. Venous malformation on the left of buttock, increased keratinization on the surface; **e** Hyperkeratosis and vascular proliferation and dilation in lesional skin by HE staining (100 ×); **f** IHC staining (100 ×) of skin lesion indicated CD31 positivity. **g** Patient P62, a girl at the age of 2 years and 11 months, had MAP3K3 p.E387D mutation. Venous malformation on right lower limb; **h** Vascular proliferation and dilation in lesional skin by HE staining (100 ×); **i** High T2 signal in fat inhibition (white arrows) in the skin and subcutaneous fat of the right lower limb

TEK somatic mutations were identified in 5 out of 6 VM patients, all of which were p.L914F hotspot. VM was characterized with blue-purple patches distributing on trunks and/or extremities with tortuous and expanded blue and red blood vessels obviously seen in some areas (Fig. 4a–c).

### VVM

We identified somatic MAP3K3 c.1323C > G;p.I441M mutation, which was previously identified in several VVM patients [19], in two VVM patients, and a novel c.1161A > C;p.E387D mutation in one patient. The mutation p.E387D was not reported in cancer patients, general populations or previous VVM patients. In silico analysis predicted it as a deleterious mutation. All mutated patients had keratinized venous malformation (Fig. 4d–i).

### Proteus syndrome

**Fig. 5:**
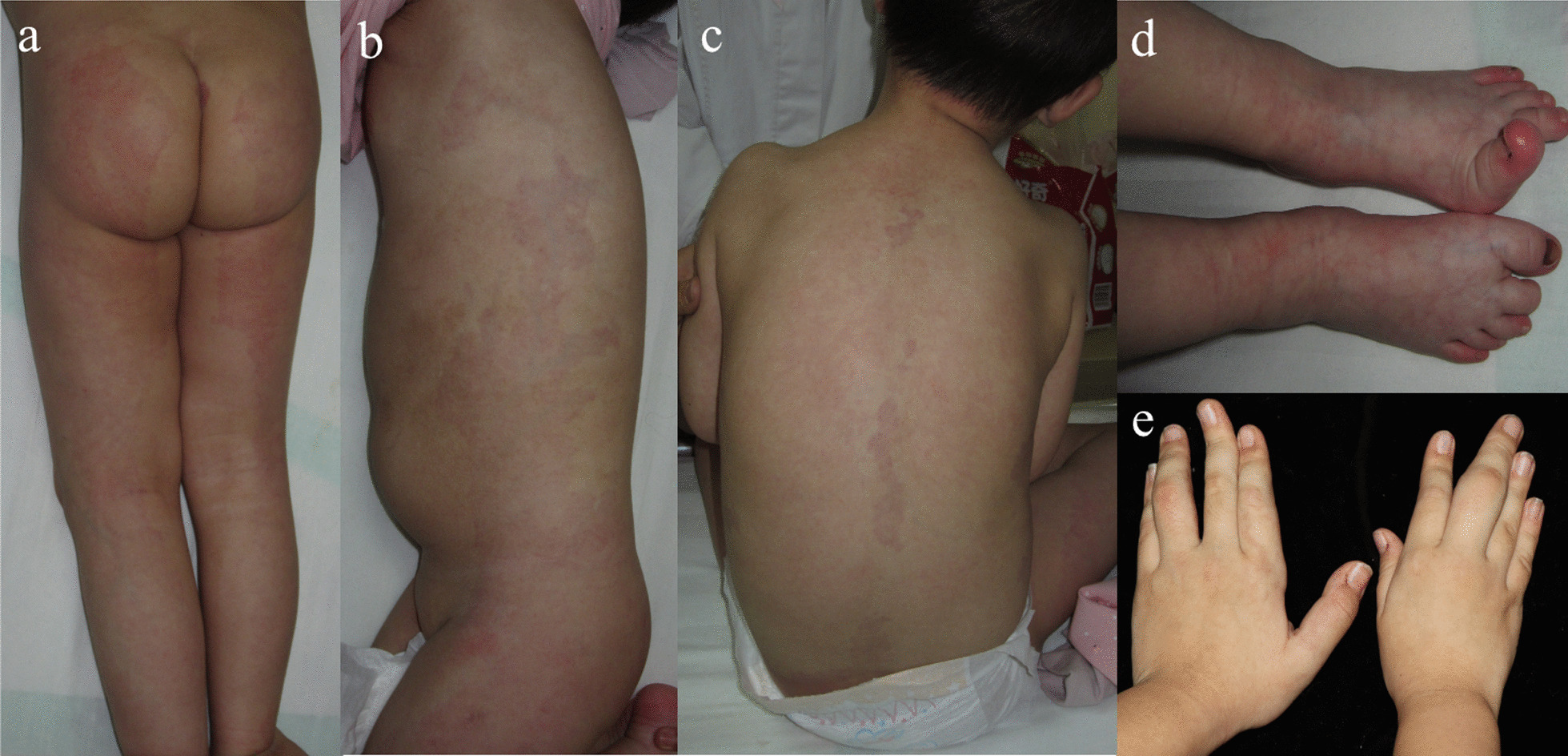
Clinical manifestations of the patient with Proteus syndrome. **a, b** Patient P5, a girl at the age of 2 years and 6 months, had dark red patches on the trunk, limbs and face with irregular geographic edges, **c** scoliosis, **d** and wide feet with widened first interdigital space. **e** Three years later when she was 5 years old, the proximal phalanges of the fourth finger of left hand and the third finger of right hand were obviously thickened with increased subcutaneous fat

Patient P5, a 2.5-year-old girl who had extensively distributed dark red patches, scoliosis and wide feet with widened first interdigital space (Fig. 5a–d), was diagnosed with Proteus syndrome. Re-examination 3 years later showed thickened fingers with increased subcutaneous fat (Fig. 5e), indicating persistent fat overgrowth. An AKT1 somatic mutation (c.235C > A;p.Q79K) was identified by WES with a VAF of 23.5%. This mutation has not been reported in previous Proteus syndrome cases. It was present in COSMIC but absent in gnomAD and was predicted as deleterious by in silico programs.

## Discussion

The present study investigated the somatic mutation spectrum of a cohort of Chinese children diagnosed with vascular malformations. Through whole-exome sequencing on skin lesion tissues with more than 200 coverage, postzygotic mutations were identified in 79.1% of patients. Our findings on the mutation spectrum in Chinese population showed that several hotspot mutations in PIK3CA, GNA11/GNAQ and TEK were responsible for most of cases, which was consistent with previous reports in other populations [8, 17, 29]. Additionally, we reported some non-hotspot, rare or novel mutations in these genes that may contribute to vascular malformations.

Somatic activating mutations of PIK3CA are important cause of various human cancers, and several hotspot mutations, including p.E542K, p.E545K and p.H1047R/L, are most frequently found [33]. Previous studies demonstrated that the majority of PIK3CA-related malformation patients, including syndromic PROS and non-syndromic ccLMs, harbored one of these hotspot mutations [10, 34–36]. These PIK3CA hotspot mutations had strong oncogenic activity characterized by several in vivo and in vitro experiments [32], and were more frequently found in PROS patients without brain overgrowth than in those with brain overgrowth [26]. In our study with 39 PIK3CA-related malformation cases, 74.4% were found to bear a PIK3CA postzygotic mutation, which were comparable with the molecular diagnostic rate of 75.5% reported by Brouillard and colleagues [31]. However, PIK3CA hotspot mutations were found in 58.6% of our cohort and even lower in PROS patients. This proportion was significantly lower than those from the other reports [10, 34–36]. Our results suggested that mutation screening of the entire PIK3CA gene, instead only detecting several hotspot mutations, may be more efficient for the molecular diagnosis in Chinese population. Apart from hotspot variants, we identified non-hotspot mutations in more than forty percent of patients with PIK3CA-related malformations, which are less frequent, have moderate oncogenic activity or have not been comparatively investigated for driver activity. A large-scale study comprising 153 patients revealed significantly different distribution of hotspot and non-hotspot mutations between PROS and ccLMs [31]. Whereas, the present study did not detect the genotype–phenotype correlation, which may be due to insufficient samples.

Human GNA11 and GNAQ genes encode the Gα11 and Gαq subunits of Gα protein, respectively, both of which share 90% amino acid identity [37]. Somatic mutations of GNA11/GNAQ, which excessively activate the MEK/ERK signaling pathway, are the most common cause of uveal melanoma with hotspot mutations mainly affecting Q209 and R183 residues [38, 39]. In patients with SWS and PWS, somatic p.R183Q mutation of GNAQ were found to be the major contributor [17], whereas mosaic GNA11 p.R183Q mutation was identified in atypical SWS [40]. Consistent with previous studies, the majority of PWS and SWS patients in our cohort had GNAQ p.R183Q mutation.

CMO has been previously found to be caused by either PIK3CA or GNA11/Q mutations. In our cohort, one CMO was found to bear a rare GNAQ p.R183G mutation which was only reported in several PWS cases [41–43], and 2 CMO patients had PIK3CA mutations [44]. In addition to cutaneous capillary malformation, a GNA11 activating mutation can cause overgrowth of tissue beneath skin lesion [20]. In our cohort, we detected GNA11 p.R183C mutation in two patients with CMO.

Proteus syndrome is mainly caused by the AKT1 mosaic mutation p.E17K that is located in the pleckstrin homology (PH) domain and causes overactivation of PI3K-AKT1 pathway [18]. In our study, the patient with Proteus syndrome did not have p.E17K hotspot but carried a novel p.Q79K mutation. This mutation is only found in several cancer cases according to COSMIC database and not previously reported in Proteus syndrome. Similar to p.E17K, p.Q79K mutation resides in PH domain, significantly increases the localization of AKT1 to cell surface independently of serum stimulation, and finally results in AKT1 activation via Thr308 and Ser473 phosphorylation [45, 46].

Somatic p.I441M mutation in MAP3K3 encoding mitogen-activated protein kinase kinase kinase 3 defines a subclass of cerebral cavernous malformation (CCM) [47, 48] and has been recently found to be responsible for VVM [19, 49]. In our study, we identified somatic p.I441M mutation in two VVM patient and a novel p.E387D mutation in another patient. This novel mutation is absent from cancer patients and general population and in silico predicted as deleterious variant. Both mutations are located in the catalytic domain. The p.I441M mutation enhances the activation of downstream MEK/ERK signaling cascade in vitro [47], but the activating effect of p.E387D mutation needs investigation.

The manifestations of vascular malformations are quite diverse. In our cohort, the majority were definitely diagnosed according to typical clinical features as well as molecular testing. However, patient P119 could not be classified as any subtype of PROS due to some atypical features. The patient presented diffuse capillary malformation and overgrowth that were suggestive of a DCMO diagnosis, and also had venous malformation on right upper limb as indicated by MRI. Since the patient had a somatic PIK3CA mutation, we classified the patient as uPROS.

It should be noted that some patients may be false negative for molecular diagnosis. We failed to identify pathogenic somatic mutations of known disease-causing genes in 3 DCMO, 4 VVM and other cases. We also failed to find possibly pathogenic mutations in other cancer-driver genes through exome-sequencing. The molecular diagnosis rate of our cohort may be underestimated for several reasons. Firstly, we collected and sequenced only one cutaneous lesion tissue sample for each patient. Zenner et al.found the detection rate of a pathogenic PIK3CA mutation in isolated LM patients was positively correlated with number of samples per individual available for testing [50]. Testing additional samples from fresh skin and surgical overgrowth tissue of those who are initially negative for next-generation sequencing may help to identify the pathogenic mutation. Secondly, we only performed WES method for molecular diagnosis, which may not be sensitive enough to detect very low-frequency (VAF < 1%) mosaic mutations although most of the sequencing had more than 300 coverage. Ultra-deep targeted sequencing, highly sensitive droplet digital PCR (ddPCR), and single molecule molecular inversion probes (smMIP) are alternative approaches for identifying mutations that are not detectable by WES. The combined application of these techniques has been displayed by Luks and colleagues [10], which resulted in molecular diagnosis in nearly 90% of patients with LM or LM-related syndromes.

## Conclusion

Taken together, we identified somatic mutations in 79.1% of Chinese pediatric patients with vascular malformations through next-generation sequencing of skin lesion tissue. Previous hotspot mutations are also frequent in our cohort, but PIK3CA non-hotspot mutations are found in a high proportion of patients. Molecular diagnosis is important for these pediatric patients with vascular malformations, which may help the clinical diagnosis, treatment and management.

### Supplementary Information

**Additional file 1.** **Table S1**. Molecular diagnosis of pediatric patients with vascular malformation.

**Additional file 2.** **Figure S1**. CMO patients with PIK3CA, GNA11 and GNAQ mutations. Patient P8, a 7-month-old girl with PIK3CA c.3073A>G;p.T1025A mutation. She had capillary malformations on right upper limb and chest (a, b). MRI showed subcutaneous vascular shadows (c). Patient P32, a 6-month-old boy with PIK3CA c.344G>C;p.R115P mutation. He had capillary malformation on left upper limb and the left side of back, and presented left upper limb overgrowth (d). CT coronal reconstruction and axial scan showed thickened soft tissue and mildly dilated vessels (white arrow) in left upper limb (e) and left shoulder (f). Patient P40, a 4-month-old girl with GNA11 c.547C>T;p.R183C mutation. She had capillary malformation on left neck and right upper limb, and slight overgrowth of right upper limb (g, h). Patient P15, a 5-month-old girl with GNAQ c.547C>G;p.R183G mutation. She had capillary malformation on chest, back and right upper limb, and overgrowth of right upper limb (i). MRI showed thickened subcutaneous soft tissue and vascular shadows (j).

**Additional file 3.** **Figure S2**. Clinical manifestations of the patient with unclassified PROS. Patient P119, a girl at the age of 8 years and 1 month with PIK3CA c.1133G>A;p.C378Y mutation, had diffuse capillary malformation and right upper limb overgrowth (a,b).

**Additional file 1.** **Figure S3**. Clinical manifestations of patients with DCMO. Patient P3, a boy at the age of 9 years and 1 month. He had reticulate capillary malformations on upper limbs and left lower limb (a-c). MRI showed overgrowth of soft tisses of left lower limb, but no venous or lymphatic anomalies were found (d, e). Patient P73, a boy at the age of 5 years and 7 month. He had diffuse capillary malformations on right trunk, lower and upper limbs, and overgrowth of right lower limb (f, g). MRI showed increased vascular signals on subcutaneous soft tissue of right lower limb (h).Patient P93, a girl at the age of 12 years and 5 month. She had capillary malformations on right lower and upper limbs and overgrowth of right lower limb (i, j). MRI showed flocculent, high-density shadows on subcutaneous soft tissue of right lower limb (k).

## Data Availability

The data that support the findings of this study are not openly available according to the Regulation of the People's Republic of China on the Administration of Human Genetic Resources and are available from the corresponding author upon reasonable request.

## Abbreviations

PROS: PIK3CA-related overgrowth spectrum
KTS: Klippel–Trenaunay syndrome
CLOVES: Congenital lipomatous overgrowth, vascular malformations, epidermal nevi, and skeletal/spinal abnormalities
PWS: Port-wine stain
SWS: Sturge–Weber syndrome
DCMO: Diffuse capillary malformation with overgrowth
VVM: Verrucous venous malformation
WES: Whole-exome sequencing
VAF: Variant allele frequency
CLVM: Capillary lymphatic venous malformation
VM: Venous malformation

## References

[1] Garzon MC, Huang JT, Enjolras O, Frieden IJ (2007). Vascular malformations: part I. J Am Acad Dermatol.

[2] Wassef M, Blei F, Adams D, Alomari A, Baselga E, Berenstein A (2015). Vascular anomalies classification: recommendations from the international society for the study of vascular anomalies. Pediatrics.

[3] Zhang B, Ma L (2018). Updated classification and therapy of vascular malformations in pediatric patients. Pediatr Investig.

[4] Adams DM, Wentzel MS (2008). The role of the hematologist/oncologist in the care of patients with vascular anomalies. Pediatr Clin North Am.

[5] Canaud G, Hammill AM, Adams D, Vikkula M, Kepple-Noreuil KM (2021). A review of mechanisms of disease across PIK3CA-related disorders with vascular manifestations. Orphanet J Rare Dis.

[6] Makinen T, Boon LM, Vikkula M, Alitalo K (2021). Lymphatic malformations: genetics, mechanisms and therapeutic strategies. Circ Res.

[7] Soblet J, Kangas J, Natynki M, Mendola A, Helaers R, Uebelhoer M (2017). Blue Rubber Bleb Nevus (BRBN) syndrome is caused by somatic TEK (TIE2) mutations. J Invest Dermatol.

[8] Limaye N, Wouters V, Uebelhoer M, Tuominen M, Wirkkala R, Mulliken JB (2009). Somatic mutations in angiopoietin receptor gene TEK cause solitary and multiple sporadic venous malformations. Nat Genet.

[9] Limaye N, Kangas J, Mendola A, Godfraind C, Schlogel MJ, Helaers R (2015). Somatic activating PIK3CA mutations cause venous malformation. Am J Hum Genet.

[10] Luks VL, Kamitaki N, Vivero MP, Uller W, Rab R, Bovee J (2015). Lymphatic and other vascular malformative/overgrowth disorders are caused by somatic mutations in PIK3CA. J Pediatr.

[11] Keppler-Noreuil KM, Rios JJ, Parker VE, Semple RK, Lindhurst MJ, Sapp JC (2015). PIK3CA-related overgrowth spectrum (PROS): diagnostic and testing eligibility criteria, differential diagnosis, and evaluation. Am J Med Genet A.

[12] Goss JA, Konczyk DJ, Smits P, Sudduth CL, Bischoff J, Liang MG (2020). Diffuse capillary malformation with overgrowth contains somatic PIK3CA variants. Clin Genet.

[13] Rodriguez-Laguna L, Ibanez K, Gordo G, Garcia-Mianur S, Santos-Simarro F, Agra N (2018). CLAPO syndrome: identification of somatic activating PIK3CA mutations and delineation of the natural history and phenotype. Genet Med.

[14] Kurek KC, Luks VL, Ayturk UM, Alomari AI, Fishman SJ, Spencer SA (2012). Somatic mosaic activating mutations in PIK3CA cause CLOVES syndrome. Am J Hum Genet.

[15] Riviere JB, Mirzaa GM, O'Roak BJ, Margaret B, Alcantara D, Conway RL (2012). De novo germline and postzygotic mutations in AKT3, PIK3R2 and PIK3CA cause a spectrum of related megalencephaly syndromes. Nat Genet.

[16] Nakashima M, Miyajima M, Sugano H, Limura Y, Kato M, Tsurusaki Y (2014). The somatic GNAQ mutation c.548G>A (p.R183Q) is consistently found in Sturge–Weber syndrome. J Hum Genet.

[17] Shirley MD, Tang H, Gallione CJ, Baugher JD, Frelin LP, Cohen B (2013). Sturge–Weber syndrome and port-wine stains caused by somatic mutation in GNAQ. N Engl J Med.

[18] Lindhurst MJ, Sapp JC, Teer JK, Johnston JJ, Finn EM, Peters K (2011). A mosaic activating mutation in AKT1 associated with the Proteus syndrome. N Engl J Med.

[19] Couto JA, Vivero MP, Kozakewich HP, Taghinia AH, Mulliken JB, Warman ML (2015). A somatic MAP3K3 mutation is associated with verrucous venous malformation. Am J Hum Genet.

[20] Couto JA, Ayturk UM, Konczyk DJ, Goss JA, Huang AY, Hann S (2017). A somatic GNA11 mutation is associated with extremity capillary malformation and overgrowth. Angiogenesis.

[21] Revencu N, Fastre E, Ravoet M, Helaers R, Brouillard P, Bisdorff-Bresson A (2020). RASA1 mosaic mutations in patients with capillary malformation-arteriovenous malformation. J Med Genet.

[22] Amyere M, Revencu N, Helaers R, Pairet E, Baselga E, Cordisco M (2017). Germline loss-of-function mutations in EPHB4 cause a second form of capillary malformation-arteriovenous malformation (CM-AVM2) deregulating RAS-MAPK signaling. Circulation.

[23] Nguyen V, Hochman M, Mihm MC, Nelson JS, Tan W (2019). The pathogenesis of port wine stain and sturge weber syndrome: complex interactions between genetic alterations and aberrant MAPK and PI3K activation. Int J Mol Sci.

[24] Mussa A, Leoni C, Iacoviello M, Carli D, Ranieri C, Pantaleo A *et al.* Genotypes and phenotypes heterogeneity in PIK3CA-related overgrowth spectrum and overlapping conditions: 150 novel patients and systematic review of 1007 patients with PIK3CA pathogenetic variants. *J Med Genet* 2022. 10.1136/jmedgenet-2021-108093 35256403

[25] Siegel DH, Cottrell CE, Streicher JL, Schilter KF, Basel DG, Baselga E (2018). Analyzing the genetic spectrum of vascular anomalies with overgrowth via cancer genomics. J Invest Dermatol.

[26] Kuentz P, St-Onge J, Duffourd Y, Courcet J, Carmignac V, Jouan T (2017). Molecular diagnosis of PIK3CA-related overgrowth spectrum (PROS) in 162 patients and recommendations for genetic testing. Genet Med.

[27] Yan W, Zhang B, Wang H, Mo R, Jiang X, Qin W (2021). Somatic frameshift mutation in PIK3CA causes CLOVES syndrome by provoking PI3K/AKT/mTOR pathway. Hereditas.

[28] He R, Liao S, Yao X, Huang R, Zeng J, Zhang J (2020). Klippel–Trenaunay and sturge–weber overlap syndrome with KRAS and GNAQ mutations. Ann Clin Transl Neurol.

[29] Yeung KS, Ip JJ, Chow CP, Kuong EY, Tam PK, Chan GC (2017). Somatic PIK3CA mutations in seven patients with PIK3CA-related overgrowth spectrum. Am J Med Genet A.

[30] Wang H, Xu Z, Lee B, Vu S, Hu L, Lee M (2019). Gain-of-function mutations in TRPM4 activation gate cause progressive symmetric erythrokeratodermia. J Invest Dermatol.

[31] Brouillard P, Schlogel MJ, Homayun Sepehr N, Helaers R, Queisser A, Fastre E (2021). Non-hotspot PIK3CA mutations are more frequent in CLOVES than in common or combined lymphatic malformations. Orphanet J Rare Dis.

[32] Dogruluk T, Tsang YH, Espitia M, Chen F, Chen T, Chong Z (2015). Identification of variant-specific functions of PIK3CA by rapid phenotyping of rare mutations. Cancer Res.

[33] Kandoth C, McLellan MD, Vandin F, Ye K, Niu B, Lu C (2013). Mutational landscape and significance across 12 major cancer types. Nature.

[34] Mirzaa G, Timms AE, Conti V, Boyle EA, Girisha KM, Martin B (2016). PIK3CA-associated developmental disorders exhibit distinct classes of mutations with variable expression and tissue distribution. JCI Insight.

[35] Osborn AJ, Dickie P, Neilson DE, Glaser K, Lynch KA, Gupta A (2015). Activating PIK3CA alleles and lymphangiogenic phenotype of lymphatic endothelial cells isolated from lymphatic malformations. Hum Mol Genet.

[36] Keppler-Noreuil KM, Sapp JC, Lindhurst MJ, Parker VE, Blumhorst C, Darling T (2014). Clinical delineation and natural history of the PIK3CA-related overgrowth spectrum. Am J Med Genet A.

[37] Lapadula D, Benovic JL (2021). Targeting oncogenic Galphaq/11 in uveal melanoma. Cancers (Basel).

[38] Van Raamsdonk CD, Griewank KG, Crosby MB, Garrido MC, Vemula S, Wiesner T (2010). Mutations in GNA11 in uveal melanoma. N Engl J Med.

[39] Van Raamsdonk CD, Bezrookove V, Green G, Bauer J, Gaugler L, O’Brien JM (2009). Frequent somatic mutations of GNAQ in uveal melanoma and blue naevi. Nature.

[40] Thorpe J, Frelin LP, McCann M, Pardo CA, Cohen BA, Comi AM (2021). Identification of a mosaic activating mutation in GNA11 in atypical Sturge–Weber syndrome. J Invest Dermatol.

[41] Cai R, Gu H, Liu F, Wang L, Zeng X, Yu W (2019). Novel GNAQ mutation(R183G) of port-wine stains: first case in East Asia. Int J Dermatol.

[42] Couto JA, Huang L, Vivero MP, Kamitaki N, Maclellan RA, Mulliken JB (2016). Endothelial cells from capillary malformations are enriched for somatic GNAQ mutations. Plast Reconstr Surg.

[43] Frigerio A, Wright K, Wooderchak-Donahue W, Tan OT, Margraf R, Stevenson DA (2015). Genetic variants associated with port-wine stains. PLoS ONE.

[44] Diociaiuti A, Rotunno R, Pisaneschi E, Cesario C, Carnevale C, Condorelli AG (2022). Clinical and molecular spectrum of sporadic vascular malformations: a single-center study. Biomedicines.

[45] Yi KH, Lauring J (2016). Recurrent AKT mutations in human cancers: functional consequences and effects on drug sensitivity. Oncotarget.

[46] Shi H, Hong A, Kong X, Koya RC, Song C, Moriceau G (2014). A novel AKT1 mutant amplifies an adaptive melanoma response to BRAF inhibition. Cancer Discov.

[47] Weng J, Yang Y, Song D, Huo R, Li H, Chen Y (2021). Somatic MAP3K3 mutation defines a subclass of cerebral cavernous malformation. Am J Hum Genet.

[48] Hong T, Xiao X, Ren J, Cui B, Zong Y, Zou J (2021). Somatic MAP3K3 and PIK3CA mutations in sporadic cerebral and spinal cord cavernous malformations. Brain.

[49] Schmidt BAR, El Zein S, Cuoto J, Al-Ibraheemi A, Liang MG, Paltiel HJ (2021). Verrucous venous malformation-subcutaneous variant. Am J Dermatopathol.

[50] Zenner K, Cheng CV, Jensen DM, Timms AE, Shivaram G, Bly R (2019). Genotype correlates with clinical severity in PIK3CA-associated lymphatic malformations. JCI Insight.

