# Supplementary material for: Somatic mutation spectrum of a Chinese cohort of pediatrics with vascular malformations
Source: Orphanet J Rare Dis. 2023 Sep 1;18:261. doi: 10.1186/s13023-023-02860-w (PMC10474751; doi:10.1186/s13023-023-02860-w)
Supplement: Supplementary file 1 — Additional file 1. Table S1. Molecular diagnosis of pediatric patients with vascular malformation. [file 13023_2023_2860_MOESM1_ESM.docx]

Table S1. Molecular diagnosis of pediatric patients with vascular malformation.

| Patient ID | Gender | Age at diagnosis | Diagnosis | Molecular diagnosis by WES | | | Ultra-deep sequencing validation ^a,^ ^b^ |
| --- | --- | --- | --- | --- | --- | --- | --- |
|  |  |  |  | Gene | Somatic mutation | Variant allele frequency ^a^ |  |
| P1 | Female | 5y3m | KTS | PIK3CA | c.1624G>A;p.E542K | 36/361 (10.0%) |  |
| P2 | Female | 7y5m | SWS | GNAQ | c.548G>A;p.R183Q | 11/424 (2.6%) | ND |
| P3 | Male | 9y1m | DCMO |  |  |  |  |
| P4 | Male | 2y5m | VM | TEK | c.2740C>T;p.L914F | 24/318 (7.5%) |  |
| P5 | Female | 2y6m | Proteus syndrome | AKT1 | c.235C>A;p.Q79K | 130/554 (23.5%) |  |
| P8 | Female | 7m | CMO | PIK3CA | c.3073A>G;p.T1025A | 15/341 (4.4%) | ND |
| P9 | Male | 3y8m | KTS | PIK3CA | c.1357G>A;p.E453K | 22/283 (7.8%) |  |
| P15 | Female | 5m | CMO | GNAQ | c.547C>G;p.R183G | 5/242 (2.1%) | 337/7177 (4.7%) |
| P16 | Female | 5m | PWS |  |  |  |  |
| P18 | Female | 10m | CLOVES | PIK3CA | c.1357G>A;p.E453K | 16/308 (5.2%) |  |
| P22 | Female | 1y4m | KTS | PIK3CA | c.1624G>A;p.E542K | 27/293 (9.2%) |  |
| P23 | Male | 4y7m | VVM |  |  |  |  |
| P24 | Female | 3y2m | VM | TEK | c.2740C>T;p.L914F | 30/442 (6.8%) |  |
| P28 | Female | 11m | KTS |  |  |  |  |
| P29 | Female | 4y | VVM |  |  |  |  |
| P30 | Female | 2y3m | KTS | PIK3CA | c.1624G>A;p.E542K | 30/448 (6.7%) |  |
| P31 | Female | 5y2m | PWS | GNAQ | c.548G>A;p.R183Q | 11/330 (3.3%) | ND |
| P32 | Male | 6m | CMO | PIK3CA | c.344G>C;p.R115P | 26/470 (5.5%) |  |
| P33 | Female | 6m | VM | TEK | c.2740C>T;p.L914F | 30/428 (7.0%) |  |
| P34 | Female | 5y1m | KTS | PIK3CA | c.3140A>G;p.H1047R | 12/386 (3.1%) | 234/6833 (3.4%) |
| P36 | Male | 1y | SWS | GNAQ | c.548G>A;p.R183Q | 18/444 (4.1%) | ND |
| P39 | Female | 3m | KTS | PIK3CA | c.1624G>A;p.E542K | 30/410 (7.3%) |  |
| P40 | Female | 4m | CMO | GNA11 | c.547C>T;p.R183C | 24/375 (6.4%) |  |
| P41 | Female | 3m | PWS | GNAQ | c.548G>A;p.R183Q | 11/318 (3.5%) | 4309/77559 (5.6%) |
| P44 | Female | 3m | PWS | GNAQ | c.548G>A;p.R183Q | 23/391 (5.9%) |  |
| P45 | Female | 9m | CMO |  |  |  |  |
| P46 | Female | 5m | KTS | PIK3CA | c.1633G>A;p.E545K | 49/477 (10.3%) |  |
| P48 | Female | 13y5m | PWS | GNAQ | c.548G>A;p.R183Q | 6/239 (2.5%) | 188/8528 (2.2%) |
| P49 | Male | 4m | CMO | GNA11 | c.547C>T;p.R183C | 18/334 (5.4%) |  |
| P51 | Male | 10y8m | KTS | PIK3CA | c.3140A>G;p.H1047R | 23/375 (6.1%) |  |
| P52 | Female | 2m | CLM | PIK3CA | c.1633G>A;p.E545K | 21/342 (6.1%) |  |
| P55 | Male | 6m | KTS | PIK3CA | c.2740G>A;p.G914R | 21/233 (9.0%) |  |
| P56 | Male | 6y2m | KTS | PIK3CA | c.1633G>A;p.E545K | 13/428 (3.0%) | ND |
| P57 | Female | 1m | KTS | PIK3CA | c.328_330delGAA;p.E110del | 62/866 (7.2%) |  |
| P60 | Male | 2y5m | CLVM |  |  |  |  |
| P62 | Male | 2y11m | VVM | MAP3K3 | c.1161A>C;p.E387D | 23/450 (5.1%) |  |
| P63 | Male | 2y3m | VM | TEK | c.2740C>T;p.L914F | 14/188 (7.4%) |  |
| P64 | Male | 4y2m | VVM | MAP3K3 | c.1323C>G;p.I441M | 17/232 (7.3%) |  |
| P65 | Male | 10y1m | VVM |  |  |  |  |
| P67 | Male | 5y9m | CLVM | PIK3CA | c.3140A>T;p.H1047L | 42/537 (7.8%) |  |
| P70 | Male | 11y | LM | PIK3CA | c.3140A>G;p.H1047R | 16/511 (3.1%) | ND |
| P71 | Male | 6y5m | CLVM | PIK3CA | c.3140A>G;p.H1047R | 20/849 (2.4%) | 5384/265974 (2.0%) |
| P72 | Male | 7y10m | CLVM | PIK3CA | c.1624G>A.p.E542K | 6/423 (1.4%) | 199/10169 (2.0%) |
| P73 | Male | 5y7m | DCMO |  |  |  |  |
| P75 | Male | 2y5m | VVM | MAP3K3 | c.1323C>G;p.I441M | 28/275 (10.2%) |  |
| P77 | Female | 5y8m | CLVM | PIK3CA | c.1624G>A.p.E542K | 9/411 (2.2%) | ND |
| P83 | Male | 1y10m | CLOVES | PIK3CA | c.2176G>A;p.E726K | 163/497 (33.0%) |  |
| P84 | Female | 1y1m | KTS | PIK3CA | c.1357G>A;p.E453K | 12/309 (3.9%) | 11225/177047 (6.3%) |
| P86 | Male | 1y2m | KTS | PIK3CA | c.1624G>A;p.E542K | 32/326 (9.8%) |  |
| P87 | Male | 4y5m | PWS | GNAQ | c.548G>A;p.R183Q | 20/220 (9.1%) |  |
| P89 | Female | 8y11m | CLVM | PIK3CA | c.1258T>C;p.C420R | 20/379 (5.3%) |  |
| P92 | Male | 6y9m | VVM |  |  |  |  |
| P93 | Female | 12y5m | DCMO |  |  |  |  |
| P95 | Male | 1y7m | CLOVES | PIK3CA | c.3139C>T;p.H1047Y | 85/723 (11.8%) |  |
| P96 | Female | 9m | CMO | PIK3CA | c.1049A>G;p.D350G | 52/385 (13.5%) |  |
| P97 | Female | 9y8m | KTS | PIK3CA | c.1633G>A;p.E545K | 15/329 (4.6%) | 4767/75779 (6.3%) |
| P104 | Female | 7y7m | VM | TEK | c.2740C>T;p.L914F | 17/228 (7.5%) |  |
| P105 | Male | 5y | PWS | GNAQ | c.548G>A;p.R183Q | 11/201 (5.5%) |  |
| P108 | Male | 1y | PWS | GNAQ | c.548G>A;p.R183Q | 7/186 (3.8%) | 15161/287196 (5.3%) |
| P109 | Male | 8m | PWS | GNAQ | c.548G>A;p.R183Q | 11/154 (7.1%) |  |
| P110 | Male | 6m | PWS |  |  |  |  |
| P113 | Male | 14y9m | CLOVES | PIK3CA | c.3140A>G;p.H1047R | 39/949 (4.1%) | 8190/207858 (3.9%) |
| P114 | Male | 1y4m | VM |  |  |  |  |
| P116 | Male | 11y4m | CLM |  |  |  |  |
| P119 | Female | 8y1m | uPROS | PIK3CA | c.1133G>A;p.C378Y | 17/222 (7.7%) |  |
| P126 | Female | 1y3m | PWS | GNAQ | c.548G>A;p.R183Q | 5/141 (3.5%) | 11415/214557 (5.3%) |
| P127 | Female | 3m | PWS | GNAQ | c.548G>A;p.R183Q | 13/165 (7.9%) |  |

^a^ Number of variant reads/all reads.

^b^ Only mutations with <5% VAF of WES were validated by ultra-deep sequencing.

KTS: Klippel-Trenaunay syndrome; SWS: Sturge-Weber syndrome; DCMO: diffuse capillary malformation with overgrowth; VM: venous malformation; CLOVES: congenital lipomatous overgrowth, vascular malformations, epidermal nevi, scoliosis/skeletal and spinal syndrome; PWS: port-wine stains; VVM: verrucous venous malformation; CMO: capillary malformation with overgrowth; LM: lymphatic malformation; CLVM: capillary lymphatic venous malformation; uPROS: unclassified PIK3CA-related overgrowth spectrum; CLM: capillary-lymphatic malformation; WES: whole-exome sequence; y: year; m: month. ND: not determined due to insufficient DNA sample.
