# Supplementary figures and images for: Somatic mutation spectrum of a Chinese cohort of pediatrics with vascular malformations
Source: Orphanet J Rare Dis. 2023 Sep 1;18:261. doi: 10.1186/s13023-023-02860-w (PMC10474751; doi:10.1186/s13023-023-02860-w)

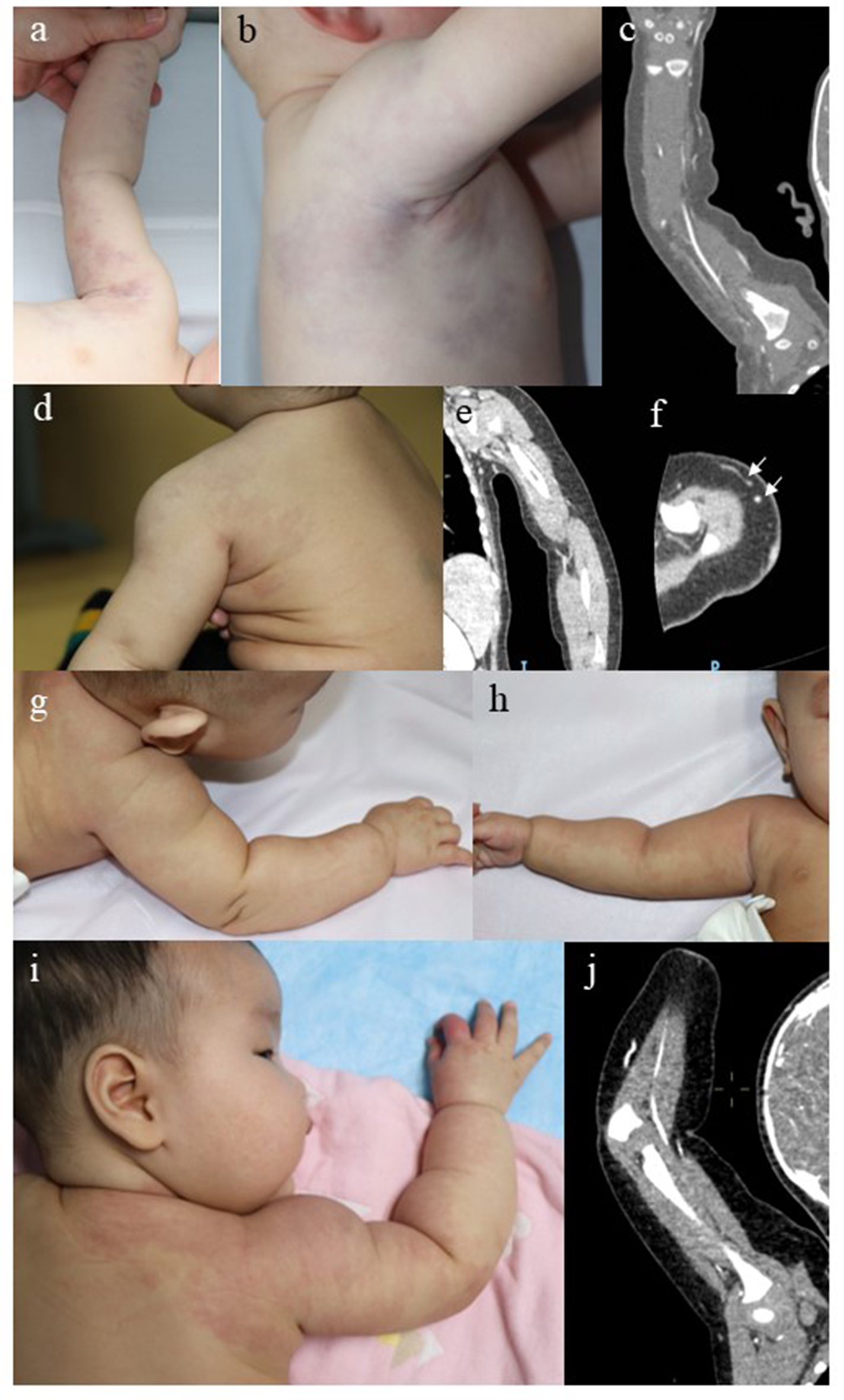

Supplement: Supplementary file 2 — Additional file 2. Figure S1. CMO patients with PIK3CA, GNA11 and GNAQ mutations. Patient P8, a 7-month-old girl with PIK3CA c.3073A>G;p.T1025A mutation. She had capillary malformations on right upper limb and chest (a, b). MRI showed subcutaneous vascular shadows (c). Patient P32, a 6-month-old boy with PIK3CA c.344G>C;p.R115P mutation. He had capillary malformation on left upper limb and the left side of back, and presented left upper limb overgrowth (d). CT coronal reconstruction and axial scan showed thickened soft tissue and mildly dilated vessels (white arrow) in left upper limb (e) and left shoulder (f). Patient P40, a 4-month-old girl with GNA11 c.547C>T;p.R183C mutation. She had capillary malformation on left neck and right upper limb, and slight overgrowth of right upper limb (g, h). Patient P15, a 5-month-old girl with GNAQ c.547C>G;p.R183G mutation. She had capillary malformation on chest, back and right upper limb, and overgrowth of right upper limb (i). MRI showed thickened subcutaneous soft tissue and vascular shadows (j). [file 13023_2023_2860_MOESM2_ESM.jpg]

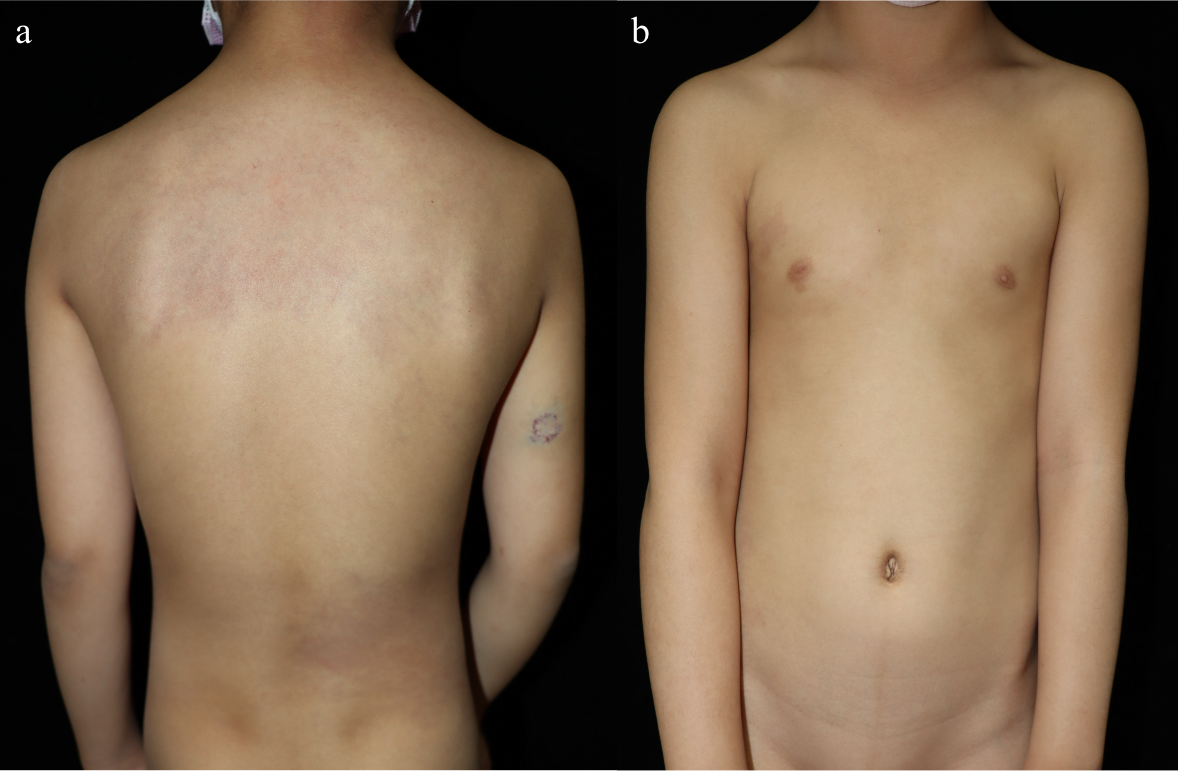

Supplement: Supplementary file 3 — Additional file 3. Figure S2. Clinical manifestations of the patient with unclassified PROS. Patient P119, a girl at the age of 8 years and 1 month with PIK3CA c.1133G>A;p.C378Y mutation, had diffuse capillary malformation and right upper limb overgrowth (a,b). [file 13023_2023_2860_MOESM3_ESM.jpg]

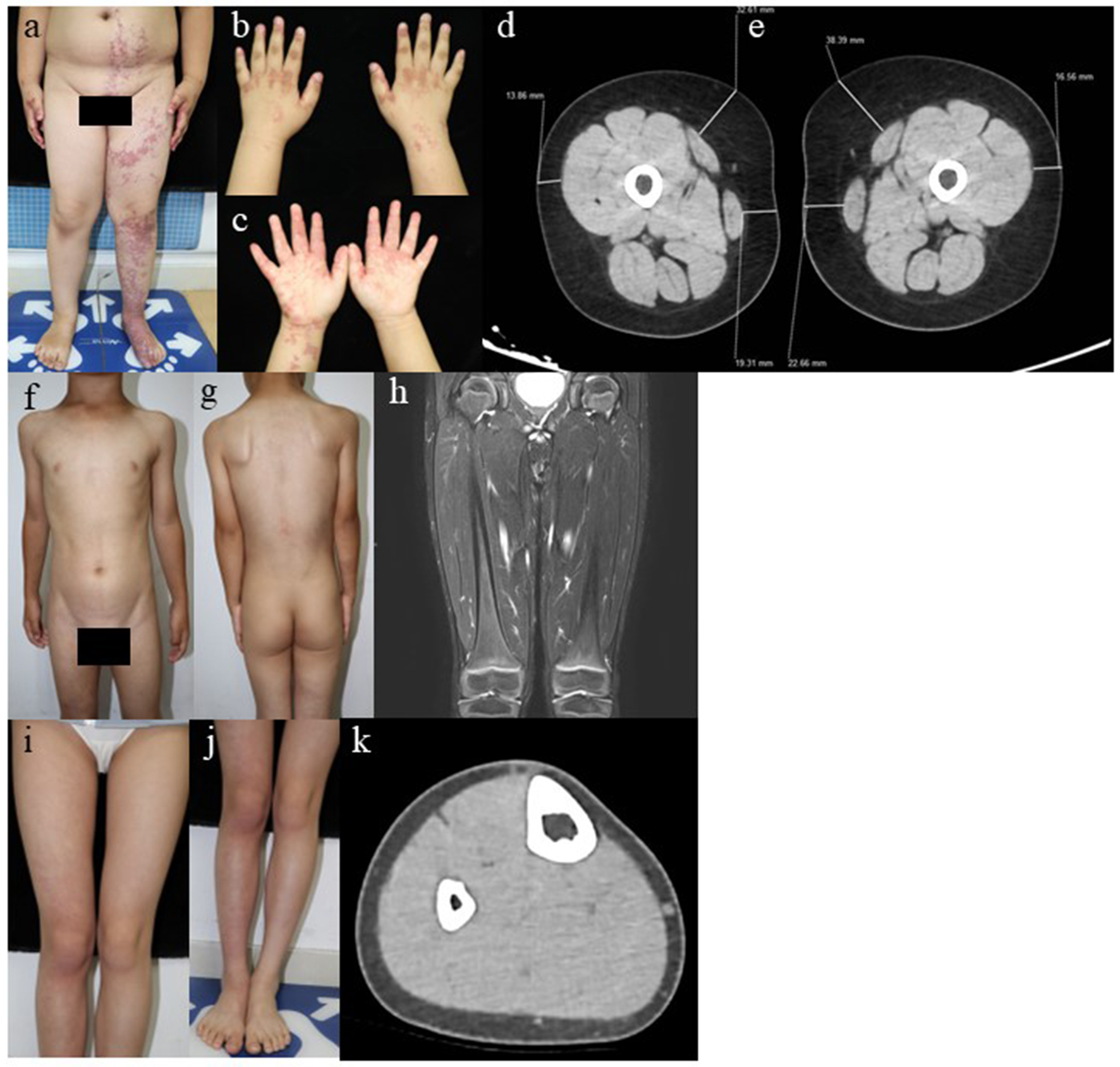

Supplement: Supplementary file 4 — Additional file 1. Figure S3. Clinical manifestations of patients with DCMO. Patient P3, a boy at the age of 9 years and 1 month. He had reticulate capillary malformations on upper limbs and left lower limb (a-c). MRI showed overgrowth of soft tisses of left lower limb, but no venous or lymphatic anomalies were found (d, e). Patient P73, a boy at the age of 5 years and 7 month. He had diffuse capillary malformations on right trunk, lower and upper limbs, and overgrowth of right lower limb (f, g). MRI showed increased vascular signals on subcutaneous soft tissue of right lower limb (h).Patient P93, a girl at the age of 12 years and 5 month. She had capillary malformations on right lower and upper limbs and overgrowth of right lower limb (i, j). MRI showed flocculent, high-density shadows on subcutaneous soft tissue of right lower limb (k). [file 13023_2023_2860_MOESM4_ESM.jpg]
